# Catastrophic Antiphospholipid Syndrome: A Rare Cause of Acute Heart Failure

**DOI:** 10.7759/cureus.42012

**Published:** 2023-07-17

**Authors:** Emad Elmusa, Muhammad Waleed Raza, Ahmad Muneeb, Hovra Zahoor, Naja Naddaf

**Affiliations:** 1 Internal Medicine, Hospital Corporation of America Florida (HCA FL) Orange Park Hospital, Orange Park, USA

**Keywords:** acute heart failure, lupus flare, sytemic lupus erythematosus, bioprosthetic, mitral valve stenosis, acute systolic heart failure, antiphospholipid syndrome, catastrophic antiphospholipid syndrome (caps)

## Abstract

Catastrophic antiphospholipid syndrome (CAPS) is a rare variant of antiphospholipid syndrome (APLS). CAPS is a syndrome characterized by microvascular thrombosis leading to multi-organ failure, including acute heart failure. Diagnosis is challenging, and disease progression is rapid. Treatment includes triple therapy with anticoagulation, glucocorticoids, and plasma exchange. We present a case of a patient with CAPS who developed de novo acute heart failure. With treatment, the patient’s multi-organ failure improved, including cardiac function. It is our goal to present this case in order to facilitate greater diagnostic suspicion and the early treatment of CAPS to reduce morbidity and mortality.

## Introduction

Approximately 40-50 people per 100,000 are diagnosed with antiphospholipid syndrome (APLS) [1]. A rare variant termed catastrophic antiphospholipid syndrome (CAPS) occurs in 1% of APLS patients [2]. The term “catastrophic” was used because of the high mortality rate associated with the syndrome when it was first described [2]. Despite current treatment, mortality remains at 30% [2]. Therefore, early diagnosis and the initiation of treatment are imperative. Diagnosis is challenging and requires high clinical suspicion. The hallmark of CAPS is microvascular thrombosis resulting in multi-organ failure, including cardiac involvement. We present a patient with CAPS who developed de novo acute heart failure. With treatment, the patient’s cardiac failure improved.

## Case presentation

A 47-year-old female with a past medical history significant for systemic lupus erythematosus (SLE), antiphospholipid syndrome, right middle cerebral artery ischemic stroke with residual left hemiparesis, type 2 diabetes mellitus, essential hypertension, chronic kidney disease stage 3B, tobacco dependence significant for 32 pack-years, and mitral valve regurgitation status post mitral valve replacement with a bioprosthetic valve five months prior presented to our facility as a transfer for the management of thrombocytopenia.

The patient’s home medications included metoprolol succinate extended release 25 mg daily, hydroxychloroquine 200 mg daily, atorvastatin 20 mg daily, insulin glargine 20 units at bedtime, and rivaroxaban 15 mg daily. Of note, the patient was taking warfarin 2.5 mg daily approximately two months prior and transitioned to rivaroxaban. The patient’s last transthoracic echocardiogram (TTE) approximately one year prior revealed an ejection fraction (EF) of 55%-60%, moderate mitral valve regurgitation, and no regional wall motion abnormalities.

The patient initially presented with a chief complaint of nausea, vomiting, and shortness of breath. Prior to transfer, the patient was diagnosed with acute chronic renal injury requiring the initiation of hemodialysis. The patient was severely thrombocytopenic, acutely hypoxic with respiratory failure requiring a 4 L nasal cannula (NC), and acutely encephalopathic. TTE revealed an ejection fraction of 20%-25%, moderate diffuse hypokinesis, and moderate to severe bioprosthetic mitral valve stenosis. The patient’s blood cultures grew oxacillin-resistant *Staphylococcus epidermidis* in two of two sets.

**Figure 1:**
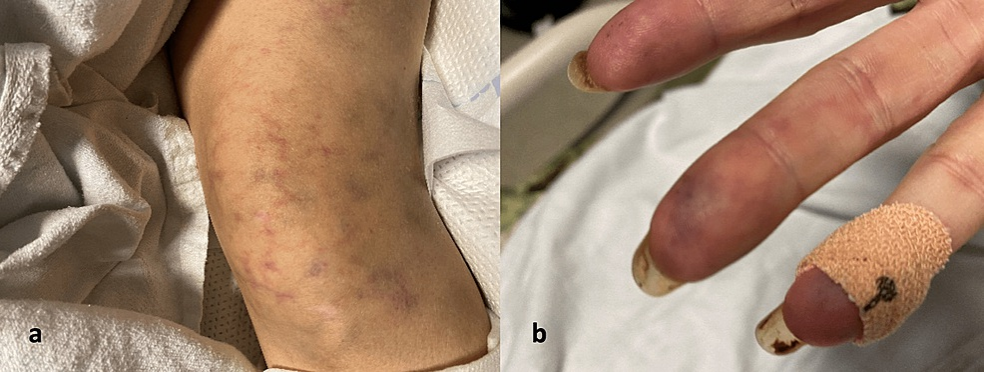
(a) Lower extremity demonstrating livedo reticularis. (b) Cyanotic fingertips.

On presentation at our facility, vitals revealed a temperature of 36.6°C, heart rate of 71 beats per minute (BPM), respiratory rate of 16 breaths per minute (BPM), blood pressure of 143/95 mmHg, and pulse oxygen saturation of 94% on a 4 L NC. Pertinent physical examination findings showed that the patient was alert and oriented only to herself, noted within the left neck was a left internal jugular vein tunneled permanent line, motor examination revealed left-sided hemiparesis, pulmonary auscultation revealed diffuse bilateral wheezing, the lower extremities demonstrated livedo reticularis (Figure 1a), and the patient’s fingertips were cyanotic and cold (Figure 1b).

**Figure 2:**
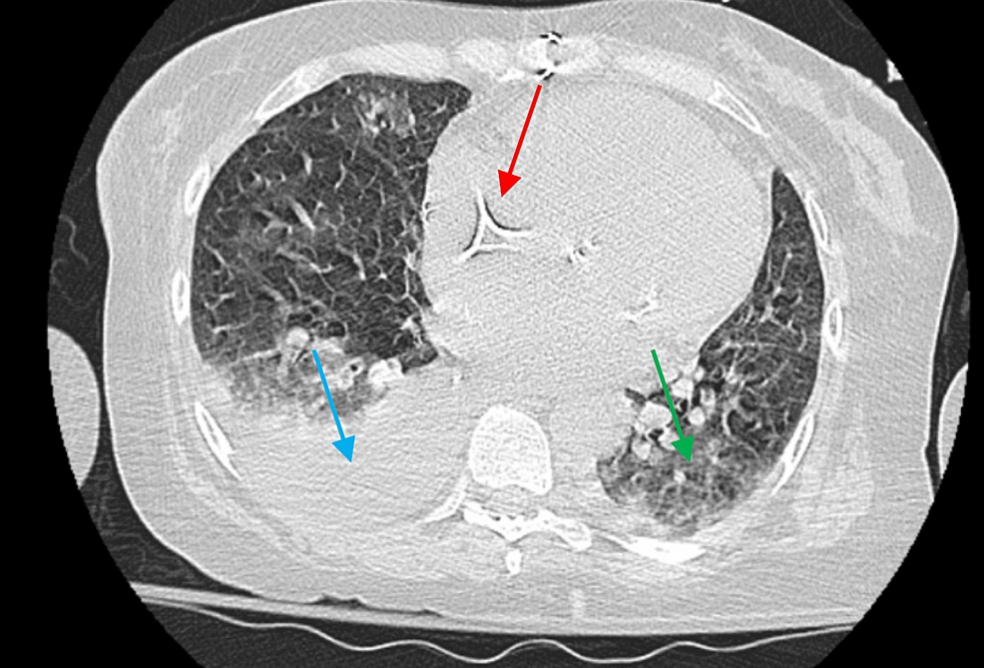
CT of the chest without contrast demonstrating right pleural effusion (blue arrow), ground-glass opacities (green arrow), and bioprosthetic mitral valve (red arrow).

**Figure 3:**
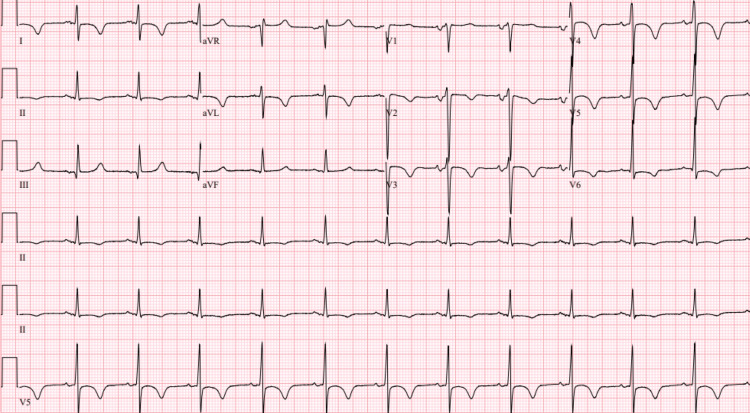
Electrocardiogram shows T wave inversions and ST changes in the antero-septal and lateral leads.

Initial laboratory results are signified in Table 1. Peripheral smear was significant for leukocytosis with absolute neutrophilia, thrombocytopenia, and rare schistocytes. CT of the chest without contrast showed bilateral pleural effusions and perihilar ground-glass opacities (Figure 2). Electrocardiogram (ECG) showed T wave inversions and ST changes in the antero-septal and lateral leads; the corrected QT (QTc) measured 497 ms (Figure 3).

**Table 1:** Laboratory values on initial presentation. INR: international normalized ratio

Laboratory test	Result	Reference range
White blood count	16.5 × 10^3^/uL	4.0-10.5 × 10^3^/uL
Hemoglobin	9.7 g/dL	11.2-15.7 g/dL
Platelet count	16 × 10^3^/uL	150-400 × 10^3^/uL
Neutrophil (%)	94.7%	34.0%-71.0%
Creatinine	3.67 mg/dL	0.55-1.02 mg/dL
Blood urea nitrogen	69 mg/dL	9-23 mg/dL
Carbon dioxide level	12.8 mEq/L	20.0-31.0 mEq/L
Anion gap	19.2 mmol/L	5-15 mmol/L
Lactic acid	0.7 mmol/L	0.4-2.0 mmol/L
Aspartate transaminase	47 U/L	0-34 U/L
Alanine transaminase	223 U/L	10-49 U/L
Ammonia	26 mcmol/L	11-35 mcmol/L
Troponin I high sensitivity	324.67 pg/mL	3-45.19 pg/mL
Brain natriuretic peptide	>5000 pg/mL	0-125 pg/mL
Prothrombin time	14.8 seconds	10.2-12.9 seconds
INR	1.28	
Activated partial thromboplastin time	38 seconds	25.1-36.5 seconds

The patient had evidence of multi-organ failure, including respiratory, renal, cerebral, and cardiac. The differential diagnosis included severe sepsis, lupus flare, microangiopathic hemolytic anemia, and CAPS. Laboratory investigation in lieu of these differentials was conducted. The patient was empirically started on triple medical therapy for concern of CAPS. The patient was started on an argatroban infusion with a goal-activated partial thromboplastin time of 60 seconds, dexamethasone 40 mg daily, and daily plasma exchange. Due to the patient’s severe thrombocytopenia, the patient’s hemoglobin and platelets were monitored every four hours. The patient’s vancomycin was continued for the treatment of bacteremia, and repeat blood cultures were negative; the duration of therapy was two weeks.

Laboratory investigations are shown in Table 2. Antiphospholipid antibodies were elevated in conjunction with multi-organ failure and physical examination findings consistent with microvascular thrombosis as diagnostic of probable CAPS. Biopsy for histopathologic analysis was deemed inappropriate given the patient’s high potential bleeding risk. Additionally, the patient was also suspected to have a lupus flare. The patient was started on rituximab 700 mg once weekly.

**Table 2:** Investigative laboratory values. ADAMTS13, a disintegrin and metalloproteinase with a thrombospondin type 1 motif, member 13; LD, lactate dehydrogenase

Laboratory test	Result	Reference range
Beta-2 microglobulin	5.58 mg/L	0.7-1.8 mg/L
Phosphatidylserine IgG antibody	74 units	0-30 units
Phosphatidylserine IgA antibody	4 units	0-19 units
Phosphatidylserine IgM antibody	33 units	0-30 units
Anti-cardiolipin IgG antibody	>112.0 GPL U/mL	<20 GPL U/mL
Anti-cardiolipin IgA antibody	>65.0 APL U/mL	<20 APL U/mL
Anti-cardiolipin IgM antibody	2.5 MPL U/mL	<20 MPL U/mL
ADAMTS13 activity	74.2%	>66.8%
Complement C3	79 mg/dL	90-180 mg/dL
Complement C4	14.60 mg/dL	14-44 mg/dL
Anti-double-strand DNA antibody	1.0 IU/mL	0-4.9 IU/mL
Iron	17 mcg/dL	50-170 mcg/dL
Total iron-binding capacity	282 mcg/dL	250-425 mcg/dL
Percent iron saturation	6.0%	
Ferritin	199 ng/mL	8-388 ng/mL
LD total	385 U/L	120-246 U/L
Haptoglobin	177.1 mg/dL	30-200 mg/dL
Fibrinogen	319 mg/dL	207-493 mg/dL
D-dimer quantitative	31825 ng/mLFEU	0.00-529 ng/mLFEU
Reticulocyte count	6.62%	0.50%-1.70%

With respect to the patient’s acute heart failure, the most probable culprit was coronary microvascular thrombosis, as well as bioprosthetic mitral valve thrombosis with stenosis in the setting of CAPS. Other possible etiologies included coronary macrovascular thrombosis, myocarditis, or infective endocarditis. The patient was not a candidate for cardiac catheterization or transesophageal echocardiography due to her severe thrombocytopenia. The patient’s troponin levels did not trend upward.

The patient’s dexamethasone was tapered down to 30 mg daily and then to 20 mg daily in a two-week span. The patient underwent plasma exchange daily for 10 days. The patient only required hemodialysis on hospital days 1 and 2.

The patient’s multi-organ failure improved, and her platelet count steadily increased (Table 3). Repeat TTE on hospital day 13 revealed an EF of 43%, a mild diffuse hypokinesis, and a bioprosthetic mitral valve without the evidence of stenosis or regurgitation.

**Table 3:** Platelet count trend.

Hospital day	Platelet count
1	16 × 10^3^/uL
2	14 × 10^3^/uL
3	26 × 10^3^/uL
4	27 × 10^3^/uL
5	52 × 10^3^/uL
6	37 × 10^3^/uL
7	33 × 10^3^/uL
8	33 × 10^3^/uL
9	34 × 10^3^/uL
10	50 × 10^3^/uL
11	56 × 10^3^/uL
12	60 × 10^3^/uL
13	49 × 10^3^/uL
14	61 × 10^3^/uL
15	59 × 10^3^/uL
16	61 × 10^3^/uL

## Discussion

Antiphospholipid antibodies bind to plasma proteins and proteins expressed or bound to vascular endothelial cells or platelets, resulting in venous and arterial thrombosis [1]. Antibodies include the lupus anticoagulant, anti-cardiolipin antibodies, or anti-B2 glycoprotein I [1]. Thrombotic complications include stroke (13%), myocardial infarction (11%), deep venous thrombosis (9.5%), and pregnancy morbidity (6%) [1].

Catastrophic antiphospholipid syndrome, or Asherson’s syndrome, is a rare variant of APLS, occurring in 1% of the patients with APLS [2]. CAPS can be precipitated by infections (49%), surgery (17%), malignancy (16%), anticoagulation withdrawal (8%), pregnancy complications (8%), drugs (5%), or SLE activity (3%) [2]. In a short time, microvascular thrombosis results in multi-organ failure. Any organ can be affected, including the kidneys, lungs, and heart [2-5]. Treatment includes triple therapy with anticoagulation, corticosteroids, and plasma exchange [2]. Despite treatment, mortality is near 30% [2]. In patients with comorbid SLE, with associated lupus flare, treatment with cyclophosphamide is indicated [2,3]. The patient’s refractory to cyclophosphamide can be placed on rituximab or eculizumab [2].

Diagnosis requires clinical evidence of multi-organ involvement, histopathologic evidence of small-vessel thrombosis, and laboratory confirmation of antiphospholipid antibodies in high titers [1]. Two of the three criteria meet probable diagnosis, and three out of three is a definite diagnosis [1].

Cardiac manifestations are common, occurring in 50% of the patients with CAPS [2]. Complications include heart failure (42.1%), valvular defects (28%), and myocardial infarction (27.8%) [2,3].

We presented a patient with CAPS. The precipitating cause could be secondary to bacteremia or rivaroxaban failure. The patient had a multi-organ failure with evidence of acute de novo heart failure based on clinical examination and echocardiography. The etiology of this is likely secondary to coronary microvascular thrombosis and bioprosthetic mitral valve thrombosis with stenosis. Addressing the underlying cause of acute heart failure is pivotal, as the pathophysiology of acute heart failure is heterogenous [6]. As demonstrated, our patient’s acute heart failure improved with the treatment of the patient’s underlying CAPS.

## Conclusions

Catastrophic antiphospholipid syndrome is a rare form of APLS. It is characterized by microvascular thrombosis leading to multi-organ failure, including cardiac failure. Diagnosis is challenging, and early treatment is essential. We presented a patient with CAPS, in whom early treatment was initiated and organs demonstrating failure improved, including the resolution of cardiac function and valve integrity.
